# Lithium Sulfide–Carbon Composites via Aerosol Spray Pyrolysis as Cathode Materials for Lithium–Sulfur Batteries

**DOI:** 10.3389/fchem.2018.00476

**Published:** 2018-10-09

**Authors:** Noam Hart, Jiayan Shi, Jian Zhang, Chengyin Fu, Juchen Guo

**Affiliations:** ^1^Department of Chemical and Environmental Engineering, University of California, Riverside, Riverside, CA, United States; ^2^Materials Science and Engineering Program, University of California, Riverside, Riverside, CA, United States

**Keywords:** aerosol spray pyrolysis, nanocomposites, lithium-sulfur batteries, lithium sulfide, sulfurization

## Abstract

We demonstrate a new technique to produce lithium sulfide-carbon composite (Li_2_S-C) cathodes for lithium-sulfur batteries *via* aerosol spray pyrolysis (ASP) followed by sulfurization. Specifically, lithium carbonate-carbon (Li_2_CO_3_-C) composite nanoparticles are first synthesized via ASP from aqueous solutions of sucrose and lithium salts including nitrate (LiNO_3_), acetate (CH_3_COOLi), and Li_2_CO_3_, respectively. The obtained Li_2_CO_3_-C composites are subsequently converted to Li_2_S-C through sulfurization by reaction to H_2_S. Electrochemical characterizations show excellent overall capacity and cycle stability of the Li_2_S-C composites with relatively high areal loading of Li_2_S and low electrolyte/Li_2_S ratio. The Li_2_S-C nanocomposites also demonstrate clear structure-property relationships.

## Introduction

Lithium-sulfur (Li-S) batteries are regarded as one of the most promising electrochemical energy storage technologies due to their low cost, environmental benignity, and outstanding theoretical capacity (Wang et al., 2013; Son et al., 2015). However, despite tremendous research and development efforts, there are still a number of challenges hindering their commercialization. Among these key challenges are the polysulfides shuttle effect and high electrolyte/sulfur ratio, which are significantly magnified by the instability of the Li metal anode (Chen J. et al., 2017; Chen S. et al., 2017; Pan et al., 2018; Wu et al., 2018). Therefore, high capacity non-Li anodes, particularly those comprised of silicon-based materials, have been proposed as replacements for Li metal in Li-S batteries (Yang et al., 2010). The use of silicon anode materials would require a pre-lithiated sulfur cathode, i.e., lithium sulfide (Li_2_S). In recent years, various methods to synthesize Li_2_S-carbon composite materials have been reported, including high-energy mixing Li_2_S with carbon (Cai et al., 2012; Jha et al., 2015), chemical lithiation of S-C composites (Hwa et al., 2015), Li_2_S-C composites synthesis *via* dissolving and precipitating Li_2_S in ethanol (Wu et al., 2014a,b,c, 2015, 2016), embedding Li_2_S in carbon matrix *via* Li-nitrogen interaction (Guo et al., 2013), reaction between Li metal and carbon disulfide (Tan et al., 2017), converting LiOH to Li_2_S *via* sulfurization with H_2_S (Dressel et al., 2016), and thermal reduction of Li_2_SO_4_ by carbon (Yang et al., 2013; Kohl et al., 2015; Li et al., 2015; Yu et al., 2017; Zhang et al., 2017; Ye et al., 2018). In addition, the mechanism studies on Li_2_S activation and capacity degradation were also reported (Vizintin et al., 2017; Piwko et al., 2018). In this work, we report a new scalable method for synthesizing Li_2_S-C composites via aerosol spray pyrolysis (ASP) followed by sulfurization.

## Materials and methods

### Materials synthesis

Three lithium salts including lithium nitrate (LiNO_3_), lithium acetate (CH_3_COOLi) and lithium carbonate (Li_2_CO_3_) were used as the precursors for Li_2_S with sucrose as the precursor for carbon. Each Li salt was dissolved in deionized water with sucrose at different concentrations as listed in Table S1. The obtained solutions were used in the ASP process.

The ASP system in this study is illustrated in Figure S1. The commercial aerosol generator (TSI, Model 3076) consisting of a nebulizer and a solution reservoir is attached to a diffusion dryer followed by a tubular furnace and a filter collector. The diffusion dryer was composed by two concentric tubes: The outer tube is made of 3-inch inner diameter PVC tubing and the inner tube is made of 0.5-inch diameter steel mesh with the annular space filled with porous silica gel. The aerosol of the precursor solution was generated by the nebulizer and carried through the diffusion dryer by argon gas to desiccate the water content. The resultant dry particles were continuously carried into the tube furnace heated at 850°C to produce the Li_2_CO_3_-C nanoparticles, which are collected with a stainless-steel filter down stream outside the tube furnace.

The synthesized Li_2_CO_3_-C composite is placed in an alumina boat in a tubular furnace, followed by purging with argon for an hour. The furnace was then heated to 725°C and maintained at this temperature for 5 h under a flow of 5 vol.% H_2_S and 95 vol.% argon. After 5 h the flow gas was switched to pure argon and the furnace was cooled naturally to room temperature. The product was collected in an argon-filled glovebox due to the sensitivity of Li_2_S to moisture.

### Materials characterization

The nitrogen adsorption-desorption isotherms of the produced composite materials were obtained with a surface area and porosity analyzer (Micromeritics ASAP2020). For a particular analysis, approximately 200 mg sample was first degassed at 150°C for 3 h, then the nitrogen adsorption-desorption isotherms were measured from 0 to 1 relative pressure. The surface area was obtained with the Brunauer-Emmett-Teller (BET) method. The crystalline species in the composites were characterized by powder X-Ray diffraction (XRD, PANalytical) with a CuKα source and a scan rate of 0.11° s^−1^. Kapton tape was used to seal the Li_2_S-C composites to protect Li_2_S from reacting with the moisture in ambient environment during measurement. The morphology and microstructure of the composites were characterized by scanning electron microscopy (SEM) and transmission electron microscopy (TEM, Tecnai T12). Carbon content in the Li_2_CO_3_-C composites was measured with thermogravimetric analysis (TGA, TA Instruments). The TGA samples were held at 120°C for 30 min to remove the moisture absorbed from environment, followed by heating to 600°C at a rate of 10°C min^−1^ with an isothermal step in dry air. The carbon contents in Li_2_CO_3_-C_NitS_, Li_2_CO_3_-C_AceS_ and Li_2_CO_3_-C_CarS_ (Figure S2) are very consistent at 20.7, 22.8, and 21.2 wt.%, respectively. Assuming complete conversion from Li_2_CO_3_ to Li_2_S without carbon loss, the Li_2_S content in Li_2_S-C_NitS_, Li_2_S-C_AceS_ and Li_2_S-C_CarS_ can be estimated as 70.4, 67.8, and 69.8 wt.%, respectively. The accurate Li_2_S content in the Li_2_S-C composites is determined as follows: 100 mg Li_2_S-C was thoroughly washed 4 times using 15 mL ethanol each time in the glovebox to remove Li_2_S. The obtained carbon was weighed after dried at 120°C for 8 h in the glovebox. The Li_2_S content is 71.3 wt.% in Li_2_S-C_NitS_, 69.1 wt.% in Li_2_S-C_AceS_, and 71.6 wt.% in Li_2_S-C_CarS_, which all agree very well with the estimated values.

### Electrode preparation and cell testing

The electrode is composed of 80 wt.% of Li_2_S-C composite, 10 wt.% of carbon black additive, and 10 wt.% of polystyrene as the binder. Polystyrene was selected as the binder to avoid the use of polar solvents (both protic and aprotic), most of which dissolve Li_2_S to some extent. Instead, mesitylene (Sigma-Aldrich) was used as the solvent for polystyrene in the electrode slurry. The electrodes were coated on carbon-coated aluminum current collector (MTI Corporation) in the argon-filled glovebox, with the average loading of Li_2_S-C composite at 2 mg cm^−2^. The electrodes were dried overnight in argon glovebox at room temperature, followed by drying at 120°C for 4 h. The dried electrodes are assembled into 2032-type coin cells with lithium foil anode (99.9%, Alfa Aesar) and Celgard® 2,500 separator. The electrolyte used in this study is 1M lithium bis(trifluoromethanesulfonyl)imide (LiTFSI) solution in a mixture of 1,3-dioxolane (DOL), dimethoxyethane (DME) and 1-butyl-1-methylpyrrolidinium bis(trifluoromethanesulfonyl)imide (Pyr14TFSI) (1:3:1 by vol.) with 1.5 wt.% of LiNO_3_. The electrolyte to Li_2_S ratio (μL/mg) was kept at 10 in all coin cells testing. To activate the Li_2_S-C electrode, the first anodic scan in the cyclic voltammetry (CV) was to 3.9 V vs. Li^+^/Li, and the anodic limit in the following scans was 2.6 V vs. Li^+^/Li. Similarly, the first charge was run at a rate of 50 mA g^−1^ (with respect to Li_2_S) to a charge cutoff of 3.5 V. Subsequent cycles are run at 117 mA g^−1^ between 2.6 V and 1.8 V vs. Li^+^/Li.

## Results and discussion

**Figure 1:**
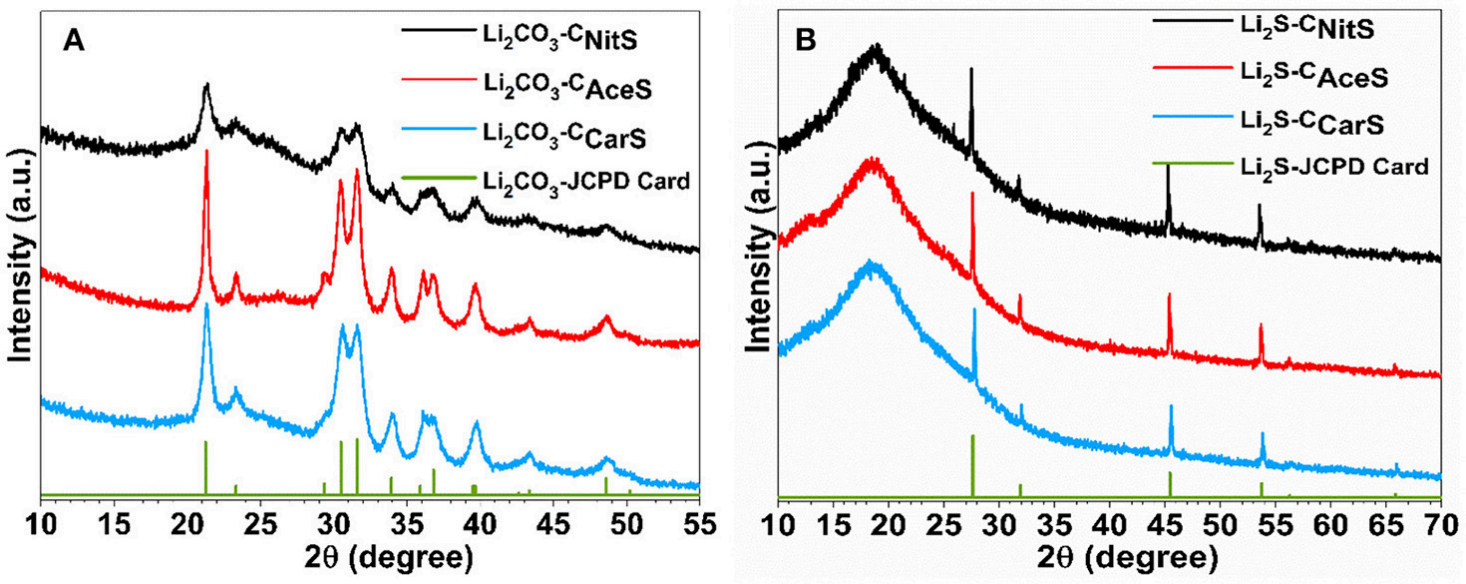
XRD patterns of **(A)** the Li_2_CO_3_-C composites obtained from ASP and **(B)** the Li_2_S-C composites after sulfurization.

During ASP synthesis, three aqueous solutions containing sucrose (as carbon precursor) and either lithium nitrate (LiNO_3_), lithium acetate (CH_3_COOLi), or lithium carbonate (Li_2_CO_3_), denoted as NitS, AceS, and CarS, respectively, were atomized into aerosols with a pressure-enabled atomizer. The aerosols were subsequently carried by argon gas through a diffusion dryer and a tubular furnace for pyrolysis within an inert environment. The powder X-ray diffraction (XRD) patterns in Figure 1A clearly indicate that the obtained composites from all three lithium salts are Li_2_CO_3_-C composite with comparable carbon content (20.7 wt.% in Li_2_CO_3_-C_NitS_, 22.8 wt.% in Li_2_CO_3_-C_AceS_ and 21.2 wt.% in Li_2_CO_3_-C_CarS_ *via* thermalgravimetric analysis, Figure S2). It is worth noting that sucrose solution without the lithium salts (i.e., precursors of Li_2_CO_3_) completely decomposes during the same ASP without any carbon formation. This observation reveals that Li_2_CO_3_ serves as the nucleation sites for carbonization of sucrose in ASP (Skrabalak and Suslick, 2006). However, the formation mechanisms of Li_2_CO_3_ from these three Li salts are clearly different. For LiNO_3_, its thermal decomposition is known to proceed according to **Reaction 1**: (Stern and Weise, 1969)

(1) $$\begin{array}{l} 2\text{L}\text{i}\text{N}{\text{O}}_{3}\to {\text{L}\text{i}}_{2}\text{O}+{2\text{N}\text{O}}_{\text{x}}+\left(2.5-\text{x}\right){\text{O}}_{2} \end{array}$$

Based on the XRD evidence of Li_2_CO_3_ with the absence of crystalline Li_2_O, it can be speculated that carbon dioxide (CO_2_) released from pyrolysis of sucrose further reacts with Li_2_O to generate Li_2_CO_3_ according to **Reaction 2**:

(2) $$\begin{array}{l} {\text{L}\text{i}}_{2}\text{O}+\text{C}{\text{O}}_{2}\to {\text{L}\text{i}}_{2}\text{C}{\text{O}}_{3} \end{array}$$

CH_3_COOLi undergoes thermal decomposition to generate Li_2_CO_3_ and acetone according to **Reaction 3**: (Roe and Finlay, 1952)

(3) $$\begin{array}{l} {2\text{C}\text{H}}_{3}\text{C}\text{O}\text{O}\text{L}\text{i}\to {\text{L}\text{i}}_{2}\text{C}{\text{O}}_{3}+{\text{C}}_{3}{\text{H}}_{6}\text{O} \end{array}$$

For the CarS precursor, Li_2_CO_3_ undergoes precipitation during ASP without decomposition, thus becoming directly embedded into the carbon matrix formed by the carbonization of sucrose.

**Figure 2:**
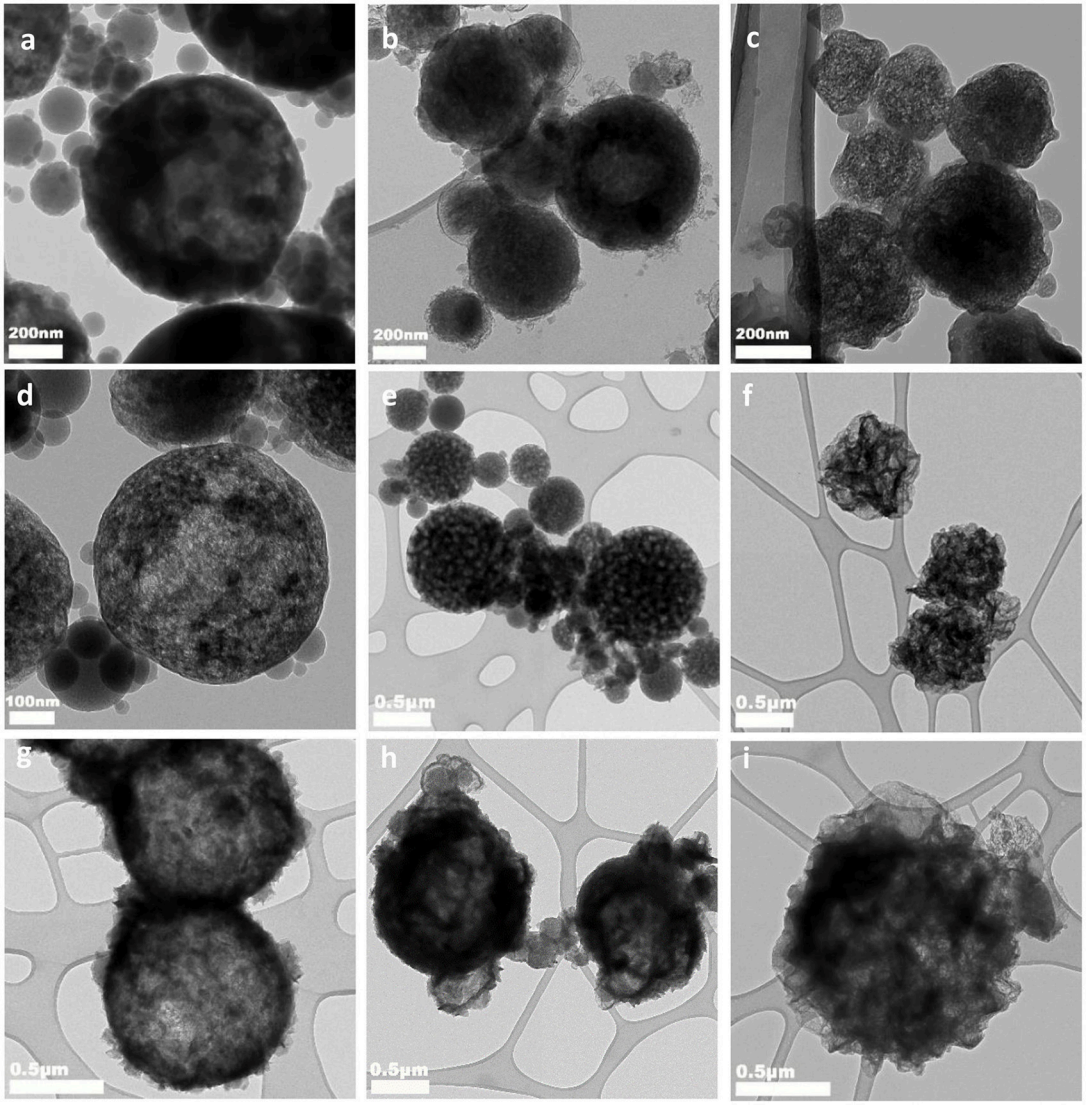
TEM images of **(a)** Li_2_CO_3_-C_NitS_, **(b)** Li_2_CO_3_-C_AceS_, **(c)** Li_2_CO_3_-C_CarS_; TEM images of the carbon matrix of **(d)** Li_2_CO_3_-C_NitS_, **(e)** Li_2_CO_3_-C_AceS_, **(f)** Li_2_CO_3_-C_CarS_ after Li_2_CO_3_ removed; and TEM images of **(g)** Li_2_S-C_NitS_, **(h)** Li_2_S-C_AceS_, **(i)** Li_2_S-C_CarS_.

**Figure 3:**
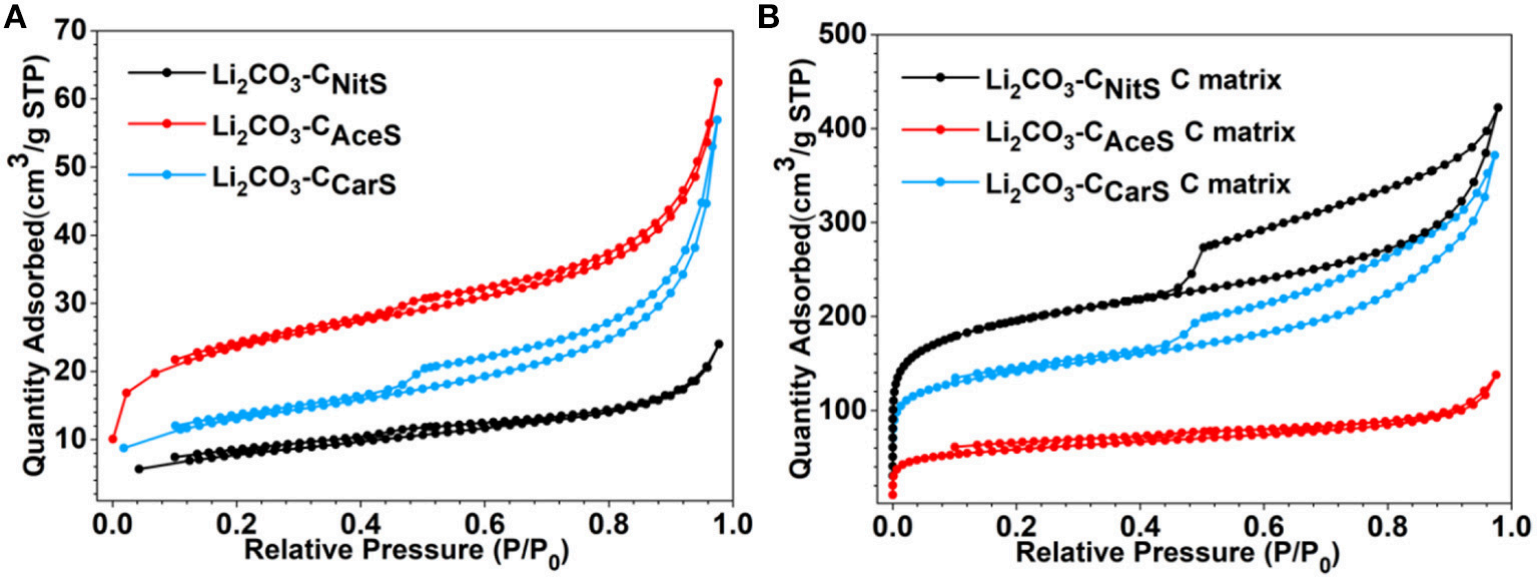
N_2_ adsorption-desorption isotherms of **(A)** Li_2_CO_3_-C nanoparticles and **(B)** the carbon matrix after Li_2_CO_3_ removal.

Although the obtained Li_2_CO_3_-C composites have consistent composition and carbon content, they have distinctively different microstructures as displayed by the transmission electron microscopy (TEM) images in Figure 2 (scanning electron microscopy images in Figure S3). The Li_2_CO_3_-C_NitS_ nanoparticles in Figure 2A have a hollow-shell structure with irregular-shaped interior voids due to the release of NO_x_ and O_2_ gases during pyrolysis. The high solubility of LiNO_3_ in water also contributes to the formation of this hollow structure. When water evaporates during ASP, LiNO_3_ precipitates at the outer surface of the aerosol droplets following the surface precipitation mechanism (Messing et al., 1993). The microstructure of the Li_2_CO_3_-C_NitS_ nanoparticles is further revealed by the TEM image in Figure 2D, after the removal of Li_2_CO_3_ using diluted hydrochloric acid (HCl). The carbon matrix of Li_2_CO_3_-C_NitS_ has a highly porous structure after Li_2_CO_3_ removal, indicating that Li_2_CO_3_ occupies the majority of the volume in the Li_2_CO_3_-C_NitS_ nanoparticles. The specific surface area of Li_2_CO_3_-C_NitS_ before and after Li_2_CO_3_ removal obtained from the nitrogen adsorption-desorption isotherms (Figure 3 and Table S2) is consistent with this observation: the specific surface area of Li_2_CO_3_-C_NitS_ is significantly increased from 26.8 to 608.2 m^2^ g^−1^ after Li_2_CO_3_ removal.

On the other hand, Li_2_CO_3_-C_AceS_ nanoparticles show a denser spherical structure in Figure 2B. It is worth noting that the AceS precursor solution has a significantly lower sucrose/lithium salt molar ratio at 1:15 compared to 1:1.5 in NitS and 1:1.18 in CarS. Given the 22.8 wt.% carbon content in Li_2_CO_3_-C_AceS_, it is believed the generated acetone during the pyrolysis of CH_3_COOLi must function as the major source for carbon formation. The TEM image of the carbon matrix after Li_2_CO_3_ removal in Figure 2E reveals the distribution of Li_2_CO_3_ in the Li_2_CO_3_-C_AceS_ nanoparticles is not as uniform as in Li_2_CO_3_-C_NitS_. The carbon matrix has a golf ball-like structure with relatively large pores, previously occupied by Li_2_CO_3_, distributed within. The specific surface area of Li_2_CO_3_-C_AceS_ is 76.3 m^2^ g^−1^, which increases to 184.9 m^2^ g^−1^ after Li_2_CO_3_ removal. This modest increase of surface area also indicates the relatively larger size of Li_2_CO_3_ compared to that of Li_2_CO_3_-C_NitS_.

As shown in Figure 2C, the Li_2_CO_3_-C_CarS_ nanoparticles clearly have a different structure resembling crumpled spheres, which is due to the much lower solubility of Li_2_CO_3_ in water than those of LiNO_3_ and CH_3_COOLi. The concentration of Li_2_CO_3_ in the CarS precursor solution is 0.1 M, which is close to saturation (Zou et al., 2013). Therefore, Li_2_CO_3_ undergoes fast and uniform precipitation from the aerosol droplets' evaporation in ASP according to the volume precipitation mechanism (Messing et al., 1993). In addition, the ASP of CarS precursor also releases fewer gaseous species without decomposition of Li_2_CO_3_. Both factors contribute to better confinement and more uniform distribution of Li_2_CO_3_. After Li_2_CO_3_ removal, the carbon matrix retains its original structure with apparently higher porosity as shown in Figure 2F. The specific surface area of Li_2_CO_3_-C_CarS_ nanoparticles is 43.7 m^2^ g^−1^, which increases to 443.6 m^2^ g^−1^ after Li_2_CO_3_ removal.

The Li_2_CO_3_-C nanoparticles obtained *via* ASP were subsequently reacted with mixed hydrogen sulfide and argon gas (H_2_S/Ar at 5/95 vol.%) at 725°C to yield the Li_2_S-C composites according to **Reaction 4**, confirmed by the XRD patterns shown in Figure 1B.

(4) $$\begin{array}{l} {\text{L}\text{i}}_{2}\text{C}{\text{O}}_{3}+{\text{H}}_{2}\text{S}\to {\text{L}\text{i}}_{2}\text{S}+{\text{H}}_{2}\text{O}+{\text{C}\text{O}}_{2} \end{array}$$

The TEM images of the Li_2_S-C composites in Figures 2G–I (scanning electron microscopy images in Figure S4) demonstrate that these nanoparticles sustain their original structures after the conversion to Li_2_S from Li_2_CO_3_.

**Figure 4:**
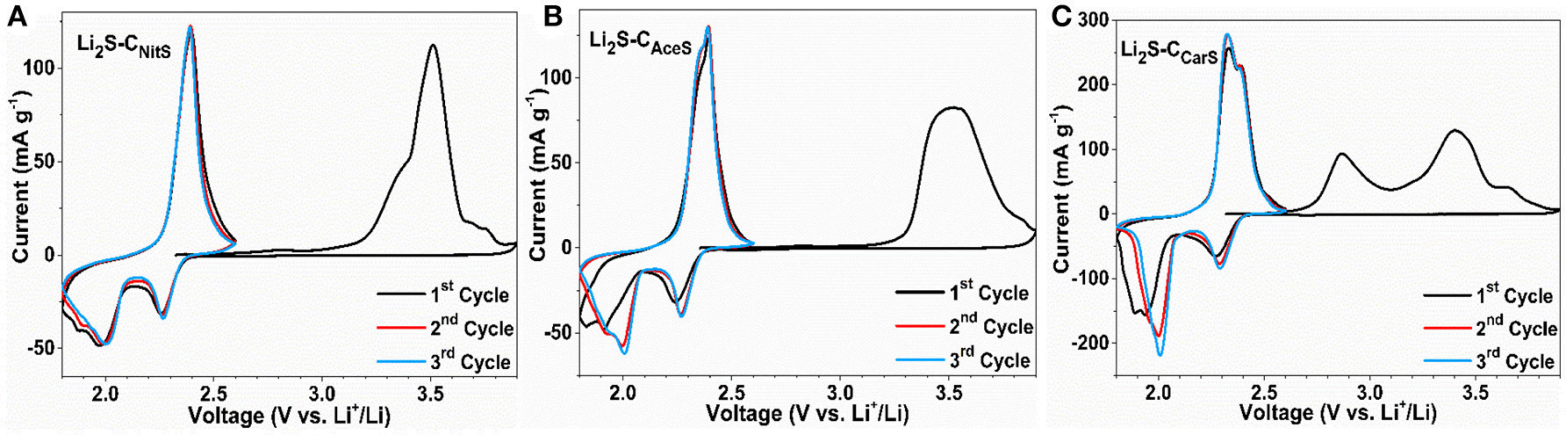
CV scans of **(A)** Li_2_S-C_NitS_, **(B)** Li_2_S-C_AceS_, **(C)** Li_2_S-C_CarS_ using 0.2 mV s^-1^ scan rate.

**Figure 5:**
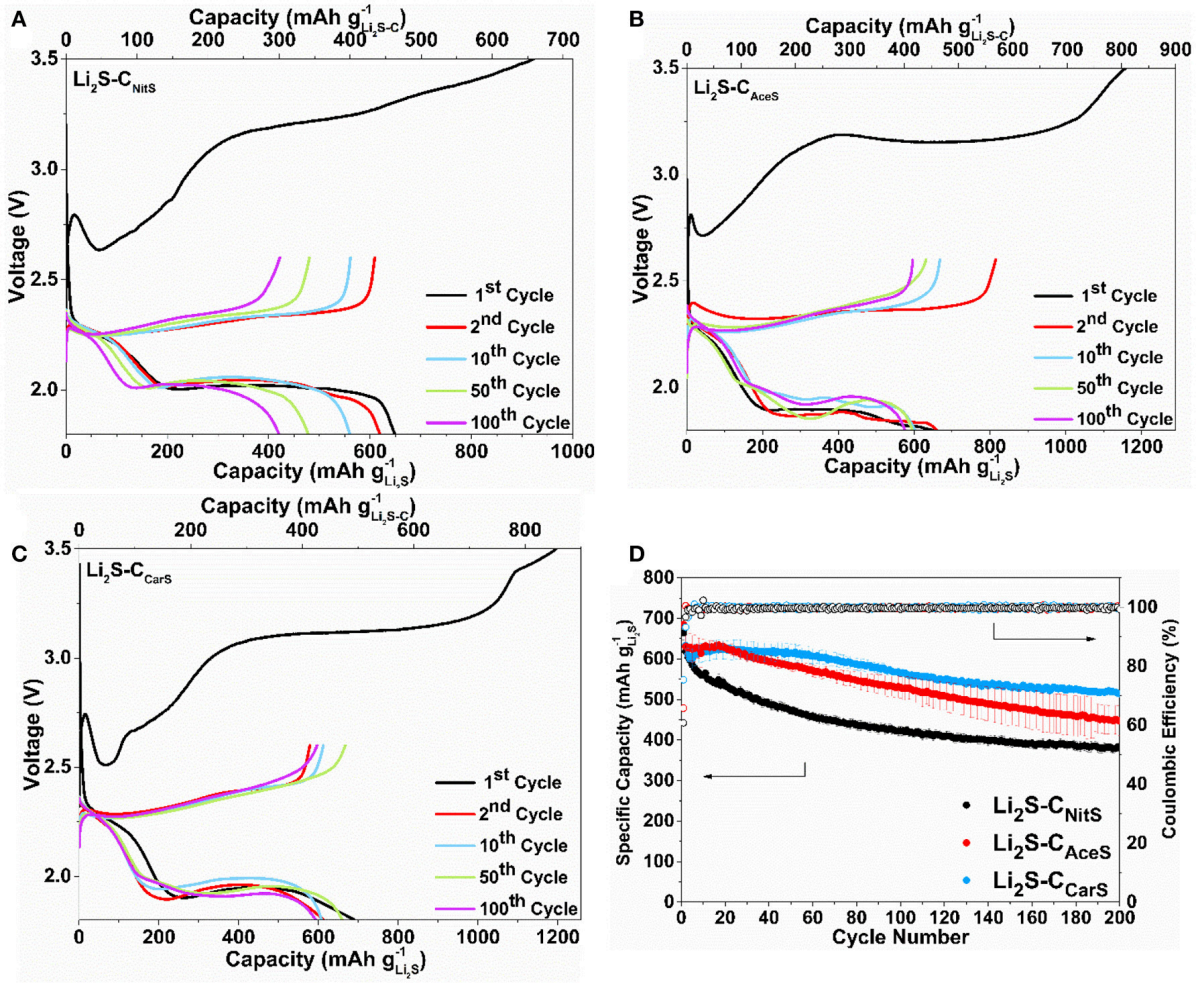
Representative charge-discharge curves of **(A)** Li_2_S-C_NitS_, **(B)** Li_2_S-C_AceS_, **(C)** Li_2_S-C_CarS_ at the 1st, 2nd, 10th, 50th, and 100th cycle, and **(D)** the cycle stability of these composites at 117 mA g^-1^.

Figure 4 shows the first three CV cycles of the Li_2_S-C vs. Li counter/reference electrode in two-electrode cells. The cathodic peak in the first delithiation scan of Li_2_S-C_NitS_ is centered at 3.5 V with a small shoulder at 3.4 V. The Li_2_S-C_AceS_ composite demonstrates a broader delithiation peak at the same potential. In contrast, Li_2_S-C_CarS_ shows two distinct cathodic peaks at 2.75 and 3.4 V vs. Li^+^/Li. The lower cathodic peak of the Li_2_S-C_CarS_ composite at 2.75 V indicates a lower energy barrier for the delithiation reaction (Zhou et al., 2017). The Li_2_S-C_CarS_ composite also demonstrates the highest peak current in the consecutive lithiation-delithiation scans. The superior performance of Li_2_S-C_CarS_ may be reflective of the intimate contact of Li_2_S and the carbon matrix. Figure 5 displays the representative charge-discharge curves and the cycle stability of the Li_2_S-C composites. The electrolyte/Li_2_S ratio is 10:1 (μL/mg), and all Li_2_S-C composites are first charged to 3.5 V (activation) vs. Li^+^/Li with a current density of 50 mA g^−1^. The charge-discharge curves demonstrate similar cycling behavior of these three Li_2_S-C composites. However, Li_2_S-C_AceS_ shows the highest charge-discharge hysteresis, which is consistent with the lowest surface area of its carbon matrix. On the other hand, although Li_2_S-C_NitS_ shows the lowest voltage hysteresis due to the highest surface area of its carbon matrix, its capacity rapidly fades. As a composite with the balanced microstructure, Li_2_S-C_CarS_ demonstrates the best overall performance: After 200 cycles, Li_2_S-C_CarS_ can retain a capacity of 540 mAh g^−1^, superior to 385 mAh g^−1^ of Li_2_S-C_NitS_ and 460 mAh g^−1^ of Li_2_S-C_AceS_, indicating the effectiveness of the Li_2_S-C_CarS_ composite architecture in sequestrating polysulfides. The overall performance demonstrated by Li_2_S-C_CarS_, in terms of areal loading, E/Li_2_S ratio, overall capacity, and cycle stability, is on par with the best performance reported to date (Table S3).

In summary, we examined a new synthetic route for the production of Li_2_S-C composite materials for Li-S batteries. The combination of aerosol spray pyrolysis and sulfurization has been shown to be a robust method for the conversion of various lithium salts including nitrate, acetate, and carbonate to Li_2_S-C nanocomposites using sucrose as the carbon precursor. Furthermore, the cycling performance of the Li_2_S-C composite has been found to be closely correlated to its precursor-derived microstructure. The combination of Li_2_CO_3_ and sucrose results in the Li_2_S-C composite with the best electrochemical performance, which has a non-hollow composite structure with Li_2_S uniformly embedded in the carbon matrix. The detailed mechanism of aerosol spray pyrolysis and the optimization of the composite's structure and electrochemical performance will be further investigated in our future studies.

## Author contributions

NH completed most of the experiments. JS, JZ, and CF helped with the experiments and data analysis. JG designed the experiments. All authors co-wrote the manuscript.

### Conflict of interest statement

The authors declare that the research was conducted in the absence of any commercial or financial relationships that could be construed as a potential conflict of interest.
