# Supplementary material for: Lithium Sulfide–Carbon Composites via Aerosol Spray Pyrolysis as Cathode Materials for Lithium–Sulfur Batteries
Source: Front Chem. 2018 Oct 9;6:476. doi: 10.3389/fchem.2018.00476 (PMC6190730; doi:10.3389/fchem.2018.00476)
Supplement: Supplementary file 1 [file Table_1.pdf]

# *Supplementary Material*

# Lithium Sulfide–Carbon Composites via Aerosol Spray Pyrolysis as Cathode Materials for Lithium–Sulfur Batteries

**Noam Hart[1], Jiayan Shi[1], Jian Zhang[2], Chengyin Fu[1], Juchen Guo[1,2*]**

*** Correspondence:** Juchen Guo: jguo@engr.ucr.edu

**Table S1.** Precursor combinations and concentrations.

| Sample Denotation | Lithium Salt & Conc. | Sucrose Conc. |
|---|---|---|
| NitS | 0.3 M $LiNO_3$ | 0.2 M |
| AceS | 0.3 M $CH_3COOLi$ | 0.02 M |
| CarS | 0.1 M $Li_2CO_3$ | 0.085 M |

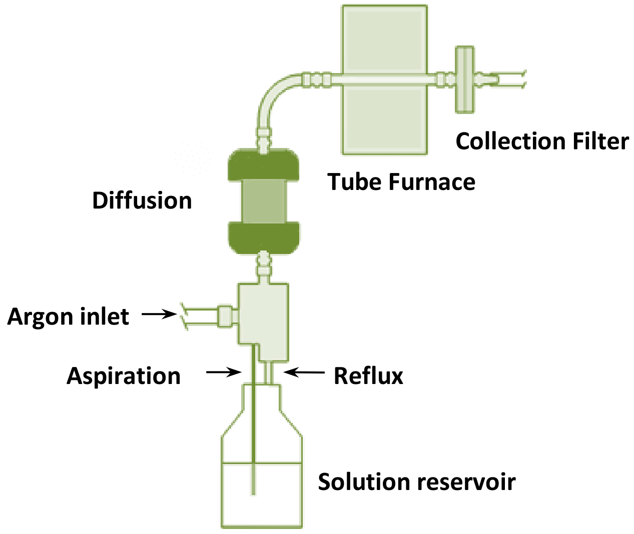


**Figure S1.** Schematic illustration of the aerosol spray pyrolysis system in this study.

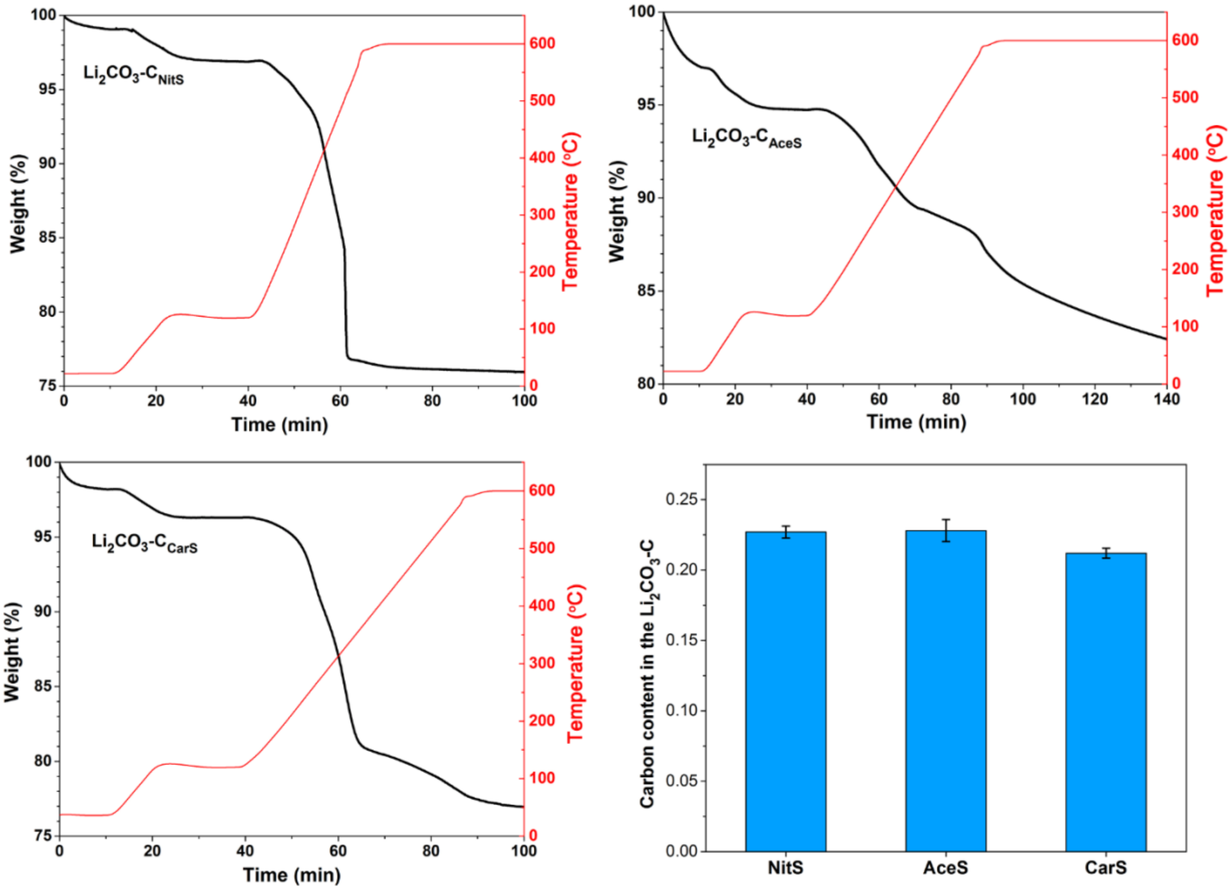


**Figure S2.** TGA of the $Li_2CO_3$-C nanocomposites.

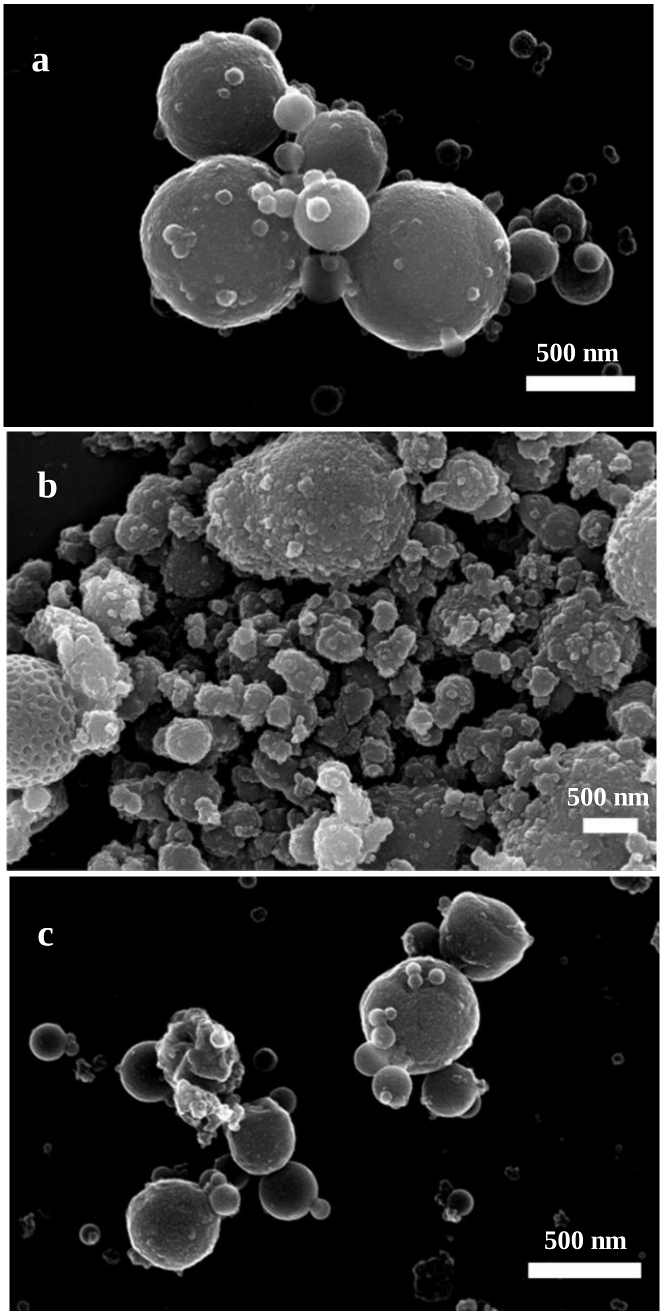


**Figure S3.** SEM images of (a) $Li_2CO_3$-$C_{NitS}$, (b) $Li_2CO_3$-$C_{AceS}$ and (c) $Li_2CO_3$-$C_{CarS}$ nanocomposites, all scale bars are **500 nm**.

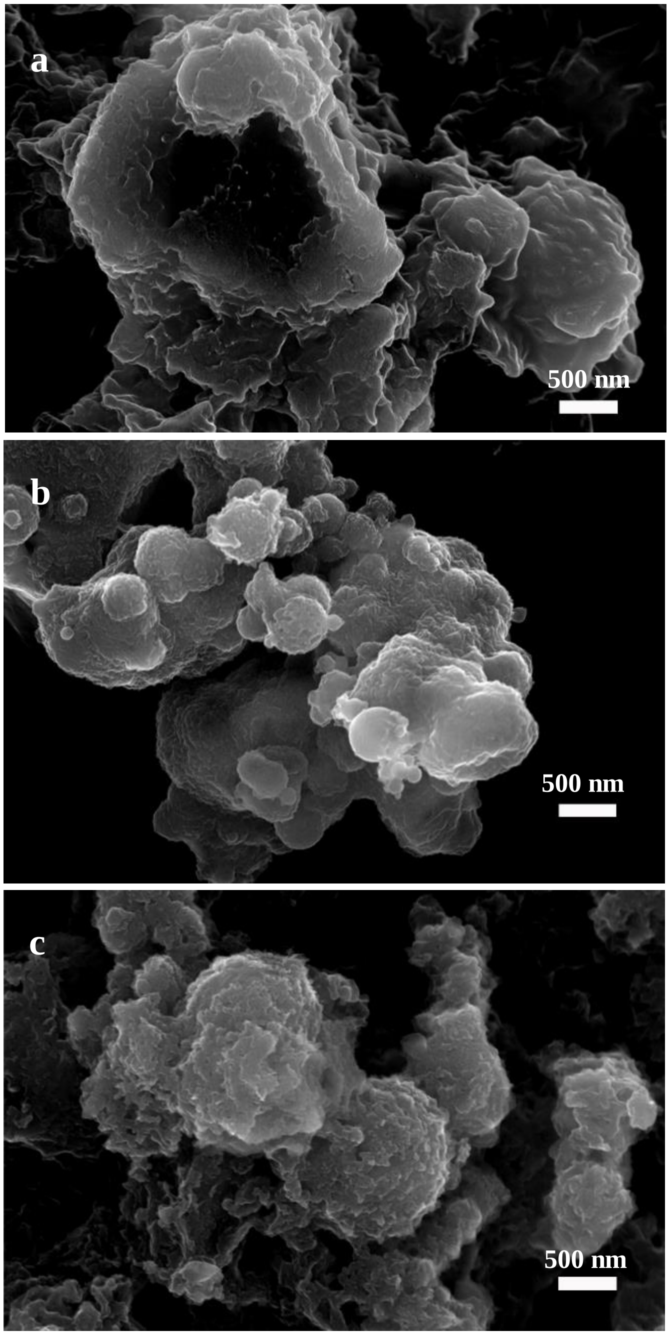


**Figure S4.** SEM images of (a) $Li_2S$-$C_{NitS}$, (b) $Li_2S$-$C_{AceS}$ and (c) $Li_2S$-$C_{CarS}$ nanocomposites, all scale bars are **500 nm**.

**Table S2.** Specific surface area of $Li_2CO_3$-C before and after $Li_2CO_3$ removal.

| Sample Name | BET Surface Area $m^2$ $g^{-1}$ | |
|---|---|---|
| | Before $Li_2CO_3$ Removal by HCl | After $Li_2CO_3$ Removal by HCl |
| $Li_2CO_3$-$C_{NitS}$ | 26.8329 | 608.2409 |
| $Li_2CO_3$-$C_{AceS}$ | 76.3216 | 184.9416 |
| $Li_2CO_3$-$C_{CarS}$ | 43.7632 | 443.6149 |

**Table S3.** Notable reports on $Li_2S$-C cathodes in literature.

| **Method** | **Areal Loading ($mg_{Li2S}$ $cm^{-2}$)** | **$E/Li_2S$ (μL:mg)** | **Capacity Retention (mAh $g^{-1}_{Li2S-C}$)** | **Cycle Number** | **Current Density (mA $g^{-1}_{Li2S}$)** |
|---|---|---|---|---|---|
| ASP in this work | 1.6 | 10:1 | 380 | 200 | 117 |
| N-butyllithium lithiated sulfur [1] | 1.1-1.4 | unavailable | 206 | 20 | 146 |
| CVD C coated Li triethylborohydride lithiated sulfur [2] | 0.7-0.9 | unavailable | 487 | 200 | 58 |
| EtOH precipitated $Li_2S$ [3] | 0.4 | unavailable | 440 | 100 | 117 |
| EtOH precipitated $Li_2S$ [4] | ~0.87 | unavailable | 609 | 100 | 233 |
| CVD C coated EtOH precipitated $Li_2S$ [5] | 1 | 118:1 | 492 | 100 | 233 |
| CVD C coated EtOH precipitated $Li_2S$ [6] | 0.6 | 196:1 | 412 | 700 | 583 |
| Thermal Reaction Li to $CS_2$ [7] | 1.6<br>4<br>8 | 10:1 | 500<br>461<br>394 | 200 | 117 |
| $Li_2SO_4$ reduction by C [8] | 0.5 | unavailable | 392 | 40 | 97 |
| $Li_2SO_4$ reduction by C [9] | unavailable | unavailable | 280 | 40 | 583 |
| $Li_2SO_4$ reduction by C [10] | ~1.24 | 26:1 | 310 | 100 | 583 |
| $Li_2SO_4$ reduction by C with Ball mill [11] | 3.4-4.5 | 3.5:1 | 283 | 150 | 117 |
| $Li_2SO_4$ reduction by C [12] | 3 | 20:1 | 316 | 50 | 233 |
| High energy milling[13] | ~0.54 | unavailable | 277 | 50 | 233 |
| $H_2S$ gas treat LiOH[14] | 2.68 | unavailable | ~410 | 100 | 233 |

REFERENCES

[1] Yang Y., McDowell T. M., Jackson A., Cha J. J., Hong S., Cui Y. (2010). New Nanostructured Li2S/Silicon Rechargeable Battery with High Specific Energy. Nano Lett. 10, 1486-1491.

[2] Hwa, Y., Zhao, J., Cairns, E. J. (2015)Lithium Sulfide (Li2S)/Graphene Oxide Nanospheres with Conformal Carbon Coating as a High-Rate, Long-Life Cathode for Li/S Cells. Nano Lett., 15, 3479-3486.

[3] Wu F., Magasinski A., Yushin G. (2014). Nanoporous Li2S and MWCNT-Linked Li2S Powder Cathodes for Lithium-Sulfur and Lithium-ion Battery Chemistries. J. Mater. Chem. A. 2, 6064–6070

[4] Wu F., Kim H., Magasinski A., Lee J., Lin H., Yushin G. (2014). Harnessing Steric Separation of Freshly Nucleated Li2S Nanoparticles for Bottom-Up Assembly of High-Performance Cathodes for Lithium-Sulfur and Lithium-Ion Batteries. Adv. Energy Mater. 4, 1400196. doi: org/10.1002/aenm.201400196.

[5] Wu F., Lee J., Fan F., Nitta N., Kim H., Magasinski A., Zhu T., Yushin G. (2015). A Hierarchical Particle ‐ Shell Architecture for Long ‐ Term Cycle Stability of Li2S Cathodes. Adv. Mater. 27, 5579 ‐ 5586. Doi.org/10.1002/adma.201502289.

[6] Wu F., Lee J., Zhao E., Zhang B., Yushin G. (2016). Graphene–Li2S–Carbon Nanocomposite for Lithium–Sulfur Batteries. ACS Nano. 10, 1333−1340.

[7] Tan G., Xu R., Xing Z., Yuan Y., Lu J., Wen J., Liu C., Ma L., Zhan C., Liu Q., Wu T., Jian Z., Yassar R., Ren Y., Miller D. J., Curtiss L. A., Ji X., Amine K. (2017). Burning lithium in CS2 for high-performing compact Li2 S–graphene nanocapsules for Li–S batteries. Nature Energy. 2, 17090

[8] Li, Z., Zhang, S., Zhang, C., Ueno, K., Yasuda, T., Tatara, R., Dokko K., Watanabe, M. (2015). One-Pot Pyrolysis of Lithium Sulfate and Graphene Nanoplatelet Aggregates: In Situ Formed Li2S/ Graphene Composite for Lithium–Sulfur Batteries. Nanoscale, 7, 14385-14392.

[9] Yang Z., Guo J., Das K. S., Yu Y., Zhou Z., Abruna D. H., Archer A. L. (2013). In Situ Synthesis of Lithium Sulfide–Carbon Composites as Cathode Materials for Rechargeable Lithium Batteries. J. Mater. Chem. A. 1, 1433.

[10] Zhang J., Shi Y., Ding Y., Peng L., Zhang W., Yu G. (2017). A Conductive Molecular Framework Derived Li2S/N,P-Codoped Carbon Cathode for Advanced Lithium ‐ Sulfur Batteries. Adv. Energy Mater. 7, 1602876. doi: org/10.1002/aenm.201602876.

[11] Kohl M., Bruckner J., Bauer I., Althues H., Kaskel S. (2015). Synthesis of Highly Electrochemically Active Li2S Nanoparticles for Lithium–Sulfur-Batteries. J. Mater. Chem. A, 3, 16307-16312.

[12] Yu M., Wang Z., Wang Y., Dong Y., Qiu J. (2017). Freestanding Flexible $Li_2S$ Paper Electrode with High Mass and Capacity Loading for High-Energy Li-S Batteries. Adv. Energy Mater. 7, 1700018. doi: 10.1002/aenm.201700018.

[13] Cai K., Song M., Cairns J. E., Zhang Y. (2012). Nanostructured $Li_2S$-C Composites as Cathode Material for High-Energy Lithium/Sulfur Batteries. Nano Lett. 12, 6474-6479

[14] Dressel B. C., Jha H., Eberle A., Gasteiger A. H., Fassler F. T. (2016). Electrochemical Performance of Lithium-Sulfur Batteries Based on A Sulfur Cathode Obtained by $H_2S$ Gas Treatment of A Lithium Salt. Journal of Power Sources. 307, 844-848
